# Systematic Determination of TCR–Antigen and Peptide–MHC Binding Kinetics among Field Variants of a *Theileria parva* Polymorphic CTL Epitope

**DOI:** 10.4049/jimmunol.2100400

**Published:** 2022-02-01

**Authors:** Nicholas Svitek, Rosemary Saya, Houshuang Zhang, Vishvanath Nene, Lucilla Steinaa

**Affiliations:** * International Livestock Research Institute, Animal and Human Health Program, Nairobi, Kenya; and; † Shanghai Veterinary Research Institute, Chinese Academy of Agricultural Sciences, Minhang District, Shanghai, China

## Abstract

CTLs are known to contribute to immunity toward *Theileria parva*, the causative agent of East Coast fever. The Tp9_67–75_ CTL epitope from the Muguga strain of *T. parva* is polymorphic in other parasite strains. Identifying the amino acids important for MHC class I binding, as well as TCR recognition of epitopes, can allow the strategic selection of Ags to induce cellular immunity toward *T. parva*. In this study, we characterized the amino acids important for MHC class I binding and TCR recognition in the Tp9_67–75_ epitope using alanine scanning and a series of variant peptide sequences to probe these interactions. In a peptide–MHC class I binding assay, we found that the amino acids at positions 1, 2, and 3 were critical for binding to its restricting MHC class I molecule BoLA-1*023:01. With IFN-γ ELISPOT and peptide–MHC class I Tet staining assays on two parasite-specific bovine CTL lines, we showed that amino acids at positions 5–8 in the epitope were required for TCR recognition. Only two of eight naturally occurring polymorphic Tp9 epitopes were recognized by both CTLs. Finally, using a TCR avidity assay, we found that a higher TCR avidity was associated with a stronger functional response toward one of two variants recognized by the CTL. These data add to the growing knowledge on the cross-reactivity of epitope-specific CTLs and specificities that may be required in the selection of Ags in the design of a wide-spectrum vaccine for East Coast fever.

## Key Points

- Positions 1–3 in the Tp9 CTL epitope are required for binding to BoLA-1*023:01.
- Positions 5–8 in the Tp9 epitope are required for TCR recognition in diverse CTLs.
- Tp9-specific CTLs from Muguga-immunized animals can cross-react with variants 4 and 7.

## Introduction

Within the adaptive immune system of vertebrate animals, CD8^+^ CTLs constitute one of the main groups of effector cells, providing a precise machinery to identify cells infected by intracellular pathogens, or cancerous cells, and eliminating them by tightly regulated mechanisms that lead to cell death (1). A critical component of CTLs that allows them to screen the host’s cell surface for foreign Ags is their TCR. The TCR is composed of two subunits, α and β, which belong to the Ig superfamily (2), and recognizes peptide epitopes bound to MHC class I molecules with a high degree of specificity (3). MHC class I molecules are heterodimeric in structure consisting of a polymorphic H chain composed of three domains (α1, α2, α3) and an invariant L chain called β_2_-microglobulin (β_2_m) (4). The α1 and α2 domains create a peptide-binding groove allowing the binding of peptides 8–11 aa residues in length in this groove, which is governed by anchor positions in the peptide sequence, while the α3 domain associates with β_2_m (5). Cytoplasmic proteins entering the MHC class I Ag processing pathway eventually result in the transport of mature peptide–MHC complexes to the cell surface and potential engagement with TCRs. Hence the amino acid sequence of a CTL peptide epitope can influence its interaction with MHC and TCR sequences and determine the induction and proliferation of a productive CTL response.

Numerous studies have demonstrated the role of CTL in long-term immunity to infectious pathogens after vaccination or infection. These include live-attenuated vaccines against the measles virus (6, 7), the yellow fever virus (8, 9), the peste des petits ruminants virus (10), influenza viruses (11), or experimental subunit vaccines developed toward HIV (12–14), *Plasmodium falciparum* (15–17), the Ebola virus (18, 19), or the severe acute respiratory syndrome or Middle East respiratory syndrome coronaviruses (20–23). In all cases, induction of CD8^+^ T cells is critical for immunity toward the pathogen, and CTL Ags have been identified for many of these pathogens for inclusion in subunit vaccines.

The intracellular apicomplexan parasite *Theileria parva*, the causative agent of East Coast fever in cattle, induces a strong CTL response and long-lived immunity, after recovery from natural infection or vaccination with a live sporozoite vaccine by an infection and treatment method (ITM) (24, 25). This method induces strain-specific immunity and can be converted into a broad-spectrum immunity by use of a mixture of sporozoites from different parasite isolates. The parasite is transmitted by the brown ear tick, *Rhipicephalus appendiculatus*, and the natural reservoir is the African buffalo, *Syncerus caffer* (26, 27). Substantial evidence indicates that CTLs induced by the ITM vaccine that lyses schizont-infected cells are the main actors providing protection to animals infected with the *T. parva* parasite. For example, it has been demonstrated that CD8^+^ T cells transferred from immune to naive animals confers passive immunity to disease, providing solid evidence that CTL Ags are candidate subunit vaccine Ags (28, 29).

Several *T. parva* CTL antigens have been identified, and some of them have been used in different experimental subunit vaccination studies resulting in partial protection toward a lethal infection by the parasite (30–33). For most of these Ags, the minimal epitope sequence was determined experimentally in the context of defined MHC class I molecules (32, 34). Recent studies have demonstrated that many of these CTL Ags vary in their amino acid sequences, which may complicate the induction of broad-spectrum vaccines (35). An example is an epitope in the Tp9 Ag from the Muguga strain of *T. parva*, which is presented by the bovine MHC class I “bovine leukocyte antigen” (BoLA)-1*023:01 molecule (36), an allele expressed in cattle of the A14 BoLA serotype. This Ag and the Tp9 epitope were shown to be polymorphic in both buffalo- and cattle-derived parasites (36). Thus, there is a concern whether the Tp9 Muguga Ag can provide cross-protection toward other field strains, especially because BoLA-1*023:01 is one of the most prevalent BoLA class I alleles in Kenya (N. Svitek, unpublished observations).

In this study, we assessed the capacity of two Tp9_Muguga_-specific CTL lines, generated from cattle of the A14 BoLA serotype immunized with the *T. parva* Muguga strain with specificity for Tp9 to cross-react with naturally occurring Tp9 epitope variants. We first aimed at identifying the minimal CTL epitope and the key anchor positions by alanine scanning of the Tp9 epitope from the Muguga strain, which determine the binding to the restricting element, the BoLA-1*023:01 class I molecule. After this, key positions important for recognition by the TCR from these CTL lines were identified, both by ELISPOT and by peptide–MHC class I tetramer (Tet) staining assays. A set of eight Tp9 peptide epitope variants was then used in BoLA-binding, ELISPOT, and peptide–MHC class I Tet staining assays to determine the level of cross-reactivity of the Tp9_Muguga_-specific CTL lines toward these variants. Finally, a novel TCR avidity assay was developed and used to compare the variation in binding strength of peptide–MHC class I Tets presenting the cross-reacting Tp9 epitope variants to the CTL lines.

## Materials and Methods

### CTL lines

Autologous *T. parva* Muguga-infected lymphocyte (TpM) lines from animals BF092, 4003, and 495 and TpM-specific bulk CTLs lines after immunization by ITM were established as previously described (37). In brief, PBMCs harvested from immunized animals were stimulated at least three times, at weekly intervals, by coculturing with irradiated autologous *T. parva*–infected cells (TpMs) at a ratio of 5–10:1 (TpMs:PBMC) in RPMI media containing 15% T cell growth factor (38). Cells were maintained in RPMI 1640 culture medium (Sigma-Aldrich) containing 10% heat-inactivated FCS (Life Technologies), 2 mM l-glutamine (Sigma-Aldrich), 1 mg/ml gentamicin (Carl-Roth), 100 UI/ml penicillin (Sigma-Aldrich), 100 mg/ml streptomycin (Sigma-Aldrich), 2-ME (BDH, final concentration: 50 μM (39); a detailed description of our protocols can be found in Ref. 40). The CTL lines from 4003 (A14 homozygous) and 495 (A10/A14 heterozygous) were propagated by stimulating them with cesium-137–irradiated autologous TpM (the use of radioisotope and irradiator was per institutional radiation safety guidelines; the length of exposure for inhibiting replication was empirically determined and found to be optimal with an exposure of 30 min at a distance of 10 cm of the cesium-137 source at 265 rad/min using a top linear irradiator [Conservatome, Lyon, France]), at a ratio of 1:5–10 (CTL:TpMs), and supplementing the media with 2 ng/ml recombinant human IL-2 (Sigma) or 15% T cell growth factor. Cells were incubated at 37°C in a 5% CO_2_ humidified atmosphere. Both CTLs recognize the Tp9 Ag. CTL clone 8 was derived from the CTL 495 by limiting dilution.

### Peptide–MHC class I binding assay

Folding of p-MHC class I complexes was assessed with an ELISA assay as described by Svitek et al. (34). The BoLA-1*023:01 molecule (25 nM) and bovine β_2_m molecule (150 nM) were incubated in a 96-well plate with Tp9_67–75_ (Muguga, G1 to A9, or V2 to V9) peptides (Mimotopes, purity: 95%) at various peptide concentrations ranging from 0 to 40 μM. After an incubation of 48 h at 18°C, the complexes were transferred to another 96-well plate precoated with streptavidin (catalog number [cat #] 436014; Nunc), which captures biotinylated heavy chains, and incubated for 3 h at 4°C. After washing, the W6/32 mAb (cat # SC-32235; Santa Cruz Biotechnology, TX), which binds to a monomorphic epitope on β_2_m, but only when it is incorporated in p-MHC class I complexes, was added for 1 h at 4°C. After washing, anti-mouse IgG coupled to peroxidase (cat # A9917-1ML; Sigma-Aldrich) was added to the plate followed by incubation for an hour at room temperature and further rounds of washes. Colorimetric change was performed by adding the TMB Plus2 “Ready to Use” Substrate (cat # 4395H; Kem En Tec) for 10 min at room temperature. The reaction was stopped by adding H_2_SO_4_ (0.3 M), and OD was measured using a Synergy HT ELISA plate reader (BioTek) at 450 nm. *K*_d_ values were determined by performing a nonlinear regression curve fit (One-Site Specific Binding) with GraphPad Prism (version 6).

### IFN-γ ELISPOT

IFN-γ ELISPOT assay was performed as previously described (34). In brief, a monoclonal anti-bovine IFN-γ Ab (cat # MCA1783; Serotec, Oxford, U.K.) was incubated overnight at 4°C on ELISPOT plates (cat # MAIPN4550; Millipore, Billerica, MA, USA) and then blocked with RPMI containing 10% heat-inactivated FCS for 2 h at 37°C. Peptides (purity: 95%; Mimotopes) were added at concentrations ranging from 0.1 to 1 μM and CD8^+^ cells at 2.5 × 10^4^ cells/well. The plates were incubated at 37°C for 20 h. Release of IFN-γ was monitored with primary rabbit polyclonal anti-bovine IFN-γ Ab (Sigma-Aldrich, St. Louis, MO, USA) and secondary alkaline phosphatase–conjugated monoclonal anti-rabbit IgG (cat # A2556; Sigma-Aldrich, St. Louis, MO). Development of plates was done by addition of the substrate solution Sigma Fast (BCIP/NBT, cat # B5655-25TAB; Sigma-Aldrich, St. Louis, MO). Plates were read using an automated ELISPOT reader (AID Classic ELISpot Reader).

### Flow cytometry

Tp9-BoLA-1*023:01 peptide–MHC class I Tets containing the Tp9_Muguga_ peptides (purity: 95%; Mimotopes) (^67^AKFPGMKKSK_76_, ^68^KFPGMKKSK_76_, ^69^FPGMKKSK_76_, ^67^AKFPGMKKS_75_, ^67^AKFPGMKK_74_), Tp9_67–75_ Muguga peptides harboring single alanine or glycine substitutions, and Tp9 peptides encoding naturally occurring epitope variants (Fig. 4A) were generated as described previously (34) and tetramerized with PE-streptavidin (cat no. 554061; BD Pharmingen) or allophycocyanin-streptavidin (cat no. 554067; BD Pharmingen). Cells were stained with 10 μl of Tp9-BoLA-1*023:01 peptide–MHC class I Tet and Abs against CD8 (IgG1, ILA51) at dilutions of 1:250. Primary Abs were labeled with secondary anti–IgG1-FITC (cat # 1070-02; Southern Biotech) at a dilution of 1:500 (25 μl per sample). All samples were stained with Fixable Viability Stain 450 (cat # 562247; BD Horizon). Staining was done in PBS–0.5% BSA. Samples were analyzed on a BD FACSCanto II flow cytometer, and data were analyzed with FlowJo (version 10). Compensation controls for PE, allophycocyanin, FITC, and Pacific Blue were included for automatic compensation by the FACSDiva software. For the analysis of CTL lines, at least 20,000 events in the lymphocytes gate were acquired after gating for live and single cells. Tet^+^ cells were either gated as a CD8^+^/Tet^+^ double-positive population or as a single population from the CD8^+^ lymphocytes population.

### TCR avidity assay

The 4003 CTL line cells were incubated in PBS 1×–0.5% BSA with 50 μM dasatinib (cat # S1021; Selleckchem) for 30 min at 37°C to prevent TCR downregulation and intracellular recycling. After this, cells were incubated with 10 μl of Tp9-BoLA-1*023:01-allophycocyanin Tets containing the Tp9_67–75_ Muguga, Tp9_V4_, or Tp9_V7_ peptides (purity: 95%; Mimotopes) and with 25 μl of anti-CD8 (IlA51) at dilutions of 1:250 for 20 min at room temperature in PBS 1×–0.5% BSA containing 50 μM dasatinib. Cells were then washed twice with PBS 1× and stained for 20 min at room temperature with 25 μl of anti-IgG1–FITC (cat #1070-02; Southern Biotech) at a dilution of 1:500 in PBS–0.5% BSA containing 50 μM dasatinib and Fixable Viability Stain 450 (cat # 562247; BD Horizon) diluted 1:1000. Next, cells were washed twice with PBS 1× and suspended in 50 μl of PBS 1×–0.5% BSA containing 50 μM dasatinib. Tp9_67–75_ Muguga-BoLA-1*023:01-PE Tets were put in excess (50 μl, equivalent to 25 nM BoLA H chain) in each well, and cells were incubated at 37°C in a humidified atmosphere containing 5% CO_2_. Cells were collected every 15 min, washed twice in PBS 1×, and fixed in 1% paraformaldehyde. This competition assay was performed for up to 165 min (total of 12 time points). Samples were analyzed on a BD FACSCanto II flow cytometer, and data were analyzed with FlowJo (version 10). Compensation controls for PE, allophycocyanin, FITC, and Pacific Blue were included for automatic compensation by the FACSDiva software. For the analysis of CTL lines, at least 25,000 events (40,000 on average) in the lymphocytes gate were acquired after gating for live and single cells. Tet^+^ cells were gated as a single population derived from the CD8^+^ lymphocytes population. Half-life (minutes) of Tet binding was assessed by measuring the percentage (%) of Tets bound at time (*t*) *x* min in the allophycocyanin-positive gate only; binding at *t* 0 min was set at 100%, and % binding at *x* min was calculated relative to % bound at *t* 0 min. Half-life was then determined by a nonlinear curve fit (dissociation − one-phase exponential decay) with GraphPad Prism (version 6). Binding was calculated as follows: % of Tp9_67–75_–Muguga-PE bound at *t* = 15 to 165 min in the PE-positive gate only minus the % of Tp9_67–75_–Muguga-PE bound on cells in that same gate at *t* = 15 min to remove cells not stained with the first Tet that could have been stained with the second competing Tet added in excess. Then, the binding (half binding time, in minutes) needed to achieve a half-maximum binding was determined by a nonlinear curve fit (one site-specific binding) with GraphPad Prism (version 6).

### Statistical analyses

Statistical analyses of data were performed with GraphPad Prism version 6 software. To determine the *K*_d_, binding, and half-life values, we performed a nonlinear regression curve fit (One-Site, Specific binding). *R*^2^ values and confidence intervals were generated to assess curve fit and differences between *K*_d_ values. When comparing data generated with the ELISPOT assay with a single peptide concentration, we performed a one-way ANOVA with Dunnett’s correction for multiple comparison. When comparing data generated with the ELISPOT assay with varying peptide concentrations, we performed a two-way ANOVA with Dunnett’s correction for multiple comparison (main column effect). All datasets were compared with the data obtained with the Tp9_67–75_ Muguga epitope. All experiments were repeated twice using duplicate measurements.

## Results

### Confirmation of the minimal Tp9 epitope for BoLA-1*023:01

In an earlier study, we had defined by ELISPOT and Tet staining a 10-mer peptide from the *T. parva* (Muguga) Tp9 Ag (^67^AKFPGMKKSK_76_) that is recognized by a CTL line from animal 495 vaccinated by ITM that kills autologous schizont-infected cells. The animal was heterozygous for BoLA-A10/BoLA-A14 and expressed the BoLA-1*023:01 allele, among other alleles expressed in this cell line. The Tp9 10-mer peptide bound to the BoLA-1*023:01 allele, and peptide–MHC class I Tets stained this CTL (34). However, the BoLA-1*023:01 allele preferentially binds 9-mer and 8-mer peptides (41), and predictions from NetMHCpan retrained on a more extensive set of peptide data predicted ^67^AKFPGMKKS_75_ as a better peptide binder to BoLA-1*023:01 (data not shown).

**FIGURE 1.**
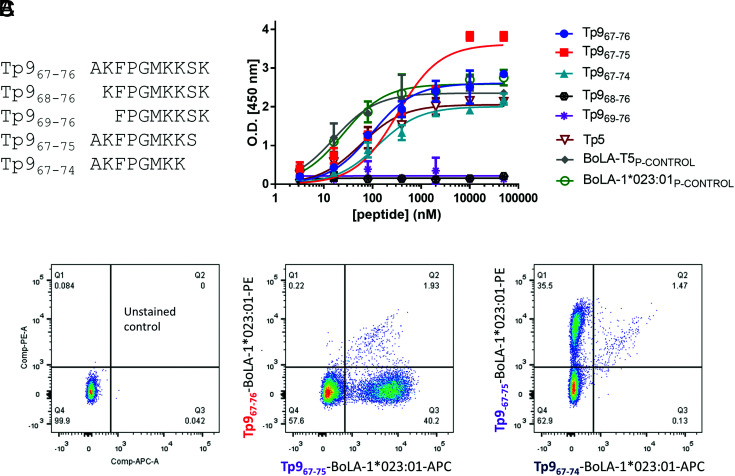
Minimal epitope sequence identification and antigenic polymorphism of the Tp9 epitope sequence. (**A**) Minimal Tp9 epitope sequences tested. (**B**) ELISA peptide-binding assay of Tp9 minimal epitopes to BoLA-1*023:01 class I molecule. (**C**) Flow cytometry staining of 495 CTL line with peptide–MHC class I Tets using BoLA-1*023:01 and Tp9_67–76_, Tp9_67–75_, and Tp9_67–74_ peptides. Mean and SEM of a representative experiment are shown. The experiments were repeated twice with duplicate measurements. BoLA-1*023:01_P-CONTROL_, WMYEGKHVL; BoLA-T5_P-CONTROL_, KMFNRTLSY; Tp5, SKADVIAKY.

We therefore decided to clarify experimentally a minimal size of the Tp9 epitope. The 10-mer and peptides with 1 or 2 aa deletions at the N- and C-terminal ends (Fig. 1A) were tested in a peptide-BoLA-1*023:01 class I molecule ELISA binding assay (Fig. 1B). An epitope from the Tp5 Ag, which was known to bind BoLA-1*023:01 class I molecule (34), was also included together with control epitopes (BoLA-T5_CONTROL_ [KMFNRTLSY] and BoLA-1*023:01_CONTROL_ [WMYEGKHVL]) not related to *T. parva*, but known to bind with high affinity to the BoLA-1*023:01 molecule. The binding assay clearly shows that deletion of 1 or 2 aa at the N-terminal end (Tp9_68–76_ and Tp9_69–76_) completely prevents peptide binding to this BoLA molecule because no folding can be measured (null OD in the ELISA), but that deletion of 1 or 2 aa at the C-terminal end does not (Tp9_67–74_ and Tp9_67–75_). These data establish the identity of the N-terminal end of the epitope, and that 10-mer, 9-mer, and 8-mer peptides can bind to BoLA-1*023:01. Similar results were obtained in IFN-γ ELISPOT using CTL 495 (Supplemental Fig. 1). An ELISPOT assay in fact confirmed that all three Tp9 peptides, which showed binding to the BoLA molecule (Tp9_67–76_, Tp9_67–75_, and Tp9_67–74_), demonstrated the capacity to stimulate a Tp9-specific CTL line (495) to similar levels (Supplemental Fig. 1). The reason that the ELISPOT data in Supplemental Fig. 1 did not exclude the 8-mer peptide as the minimal epitope is most likely due to saturation reached in the assay because relatively high peptide concentration was used in that experiment. Because it was difficult to determine which was the authentic minimal epitope recognized by this cell line, a peptide–MHC class I staining was done using combinations of Tets harboring the different 8-, 9-, and 10-mer Tp9 peptides, which were positive in the IFN-γ ELISPOT assay. This assay undoubtedly demonstrated that the most dominant minimal Tp9 epitope was in fact Tp9_67–75_ because the Tet generated with this epitope stained the majority of cells as a clearly distinct population, whereas cells stained with the other Tets were constituting only a very small fraction of this CTL line (Fig. 1C). Moreover, this exact dominant minimal 9-mer epitope sequence of the *T. parva* Muguga Tp9 Ag was independently confirmed by another group (sequence mentioned in Ref. 42).

### Determination of Tp9 epitope anchor positions using alanine scanning and glycine-substituted Tp9 peptides in peptide binding assays with BoLA-1*023:01

**FIGURE 2.**
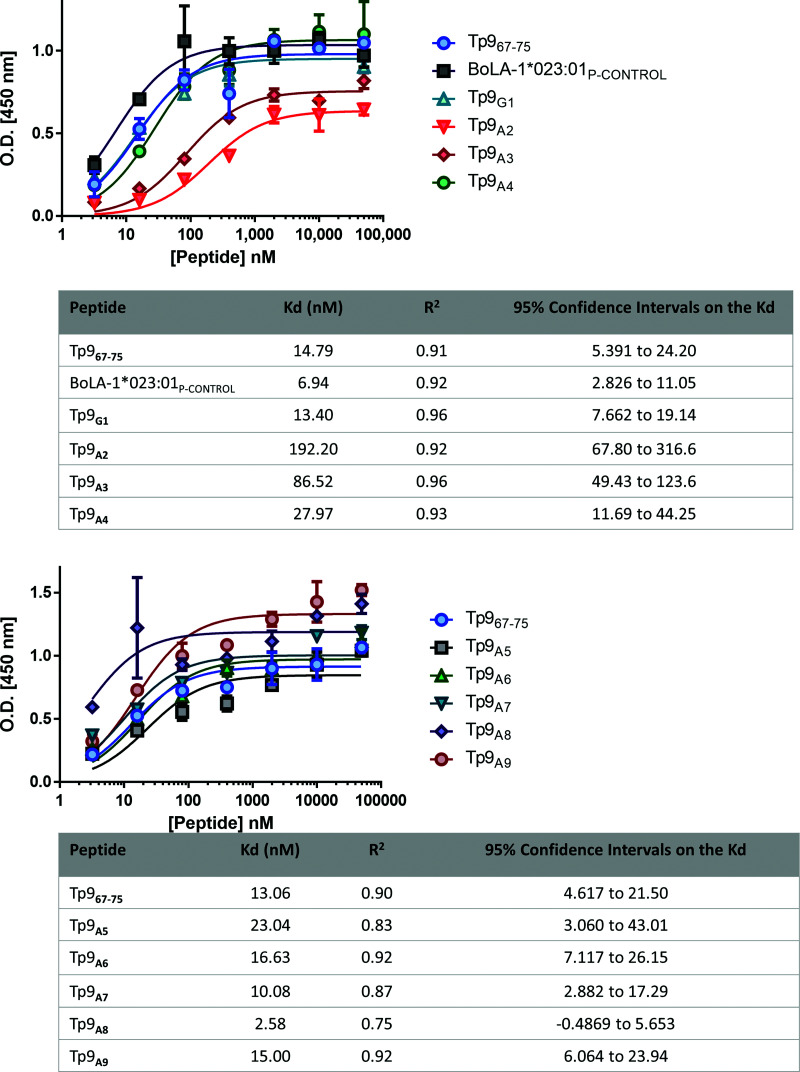
Anchor positions of the Tp9 peptide epitope. Peptide–MHC class I monomer formation ELISA assay using alanine/glycine-substituted Tp9_67–75_ epitopes. Upper panel, Tp9_67–75_ Muguga, BoLA-1*023:01 control peptide (WMYEGKHVL), Tp9_G1_, Tp9_A2_, Tp9_A3_, and Tp9_A4_. Lower panel, Tp9_67–75_ Muguga, Tp9_A5_, Tp9_A6_, Tp9_A7_, Tp9_A8_, and Tp9_A9_. *K*_d_ values (nM) are depicted in the tables below. Mean and SEM of a representative experiment are shown. The experiment was repeated twice with duplicate measurements.

A recent study demonstrated sequence diversity of the Tp9 Ag in buffalo- and cattle-derived parasites (36, 43), including in the Tp9 Muguga epitope sequence restricted by BoLA-1*023:01. To identify the key amino acids in the Tp9 epitope important for binding to the BoLA-1*023:01 MHC class I molecule, we designed a series of peptides with alanine substitutions in the Tp9_67–75_ Muguga epitope as performed with the Tp1 *T. parva* CTL epitope in a previous study (44), except for the amino acid at position 1, which was already an alanine. This was replaced by a glycine instead. The ELISA-based peptide–BoLA class I binding assay was used to determine the binding affinities of the substituted peptides. Peptides with alanine at position 2 or 3 demonstrated a substantially lower binding affinity to BoLA-1*023:01 molecule as measured by a higher *K*_d_ value (192.2 and 86.2 nM, respectively). The other substituted peptides showed binding affinities similar to the Tp9_67–75_ Muguga epitope: 13.40 nM for Tp9_G1_, 27.97 nM for Tp9_A4_, 23.04 nM for Tp9_A5_, 16.63 nM for Tp9_A6_, 10.08 nM for Tp9_A7_, 2.58 nM for Tp9_A8_, and 15.00 nM for Tp9_A9_ as compared with 13.93 nM for Tp9_67–75_ Muguga (average between experiments) and 6.94 nM for the BoLA-1*023:01–positive binding control (Fig. 2). This shows that positions 2 and 3 are key anchor residues because these alanine substitutions render them as weak binders (*K*_d_ values of at least 100% increase as compared with the control Tp9_67–75_ used in the assay and up to 500 nM are considered weak binders).

### Determination of Tp9 epitope TCR contact residues using alanine scanning and glycine-substituted Tp9 peptides in IFN-γ ELISPOT assays

**FIGURE 3.**
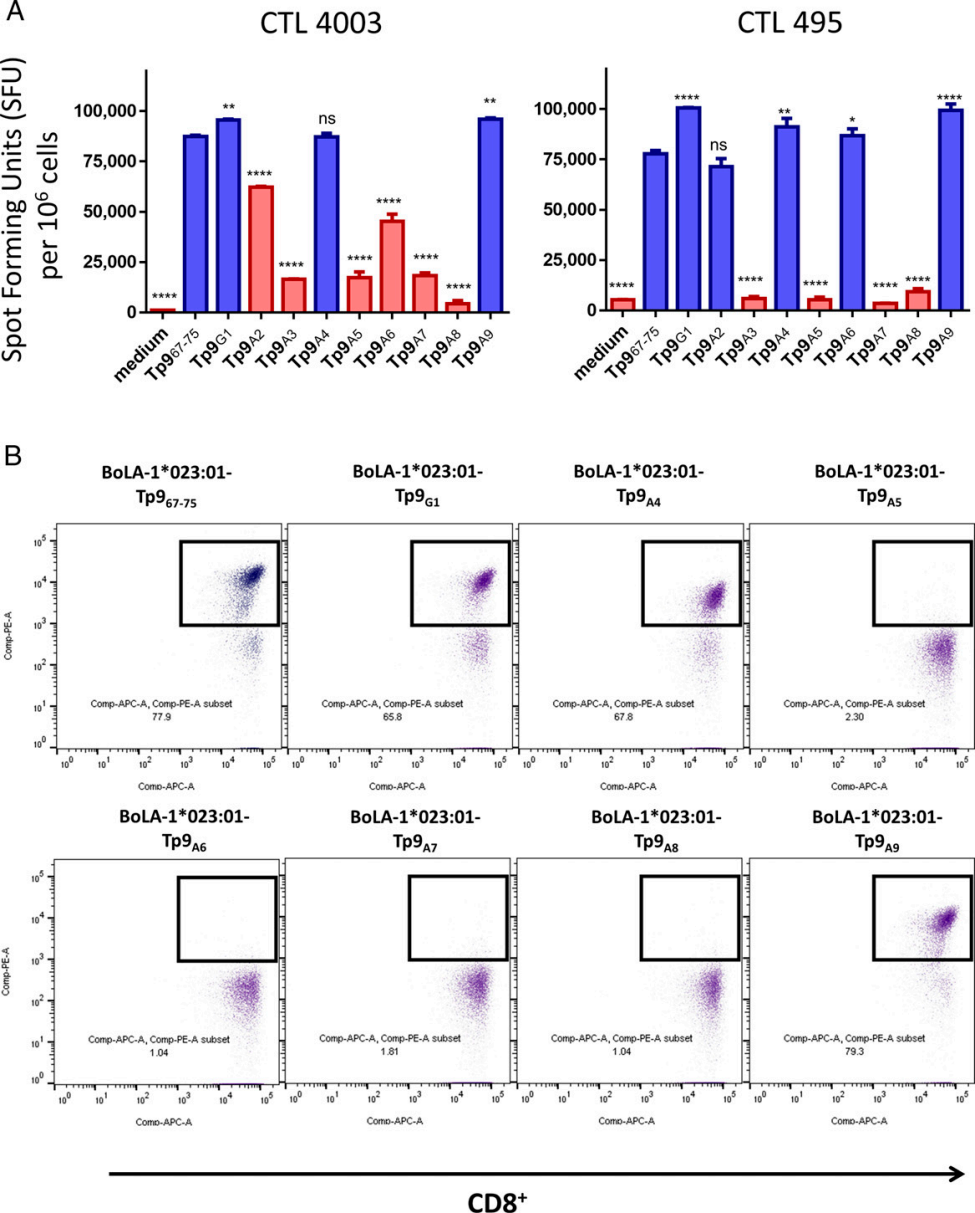
TCR recognition of the Tp9 epitope amino acids. (**A**) Two Tp9-specific CTLs (from animals 4003 and 495) were subjected to an ELISPOT assay using alanine/glycine-substituted Tp9 epitopes. Data represent spot-forming units (SFUs) per 10^6^ CD8^+^ cells. (**B**) Flow cytometry staining of CTL line 4003 with peptide–MHC class I Tets prepared with BoLA-1*023:01-Tp9_67–75_ Muguga, Tp9_G1_, Tp9_A4_, Tp9_A5_, Tp9_A6_, Tp9_A7_, Tp9_A8_, or Tp9_A9_ and anti-CD8 Ab (ILA51). Blue histogram: CTL response maintained; red histogram: CTL response reduced. Statistical analyses were done with Tp9_67–75_ as the reference group. A response was considered as reduced when the SFU was >15% lower than that of the Tp9_67–75_ SFU. *p* values (asterisks indicate level of significance), mean, and SD of a representative experiment are shown. ^ns^*p* > 0.05, *p = 0.0425, **p ≤ 0.01, ****p ≤ 0.0001. The experiments were repeated twice with duplicate measurements. ns, not significant.

The next step was to identify the key amino acids in the Tp9 epitope that influence recognition by Tp9-specific CTLs originating from two different cattle immunized by ITM. As expected, the two peptides with alanine at positions 2 (Tp9_A2_) and 3 (Tp9_A3_) showed a decreased capacity to stimulate CTL 495 and CTL 4003 for the second peptide and CTL 4003 for the first peptide (Fig. 3A). Additional variations were observed between the two CTL lines in terms of their reactivity toward the different alanine-substituted peptides. This was the case for Tp9_A6_, which reacts to lower levels with CTL 4003 but reacted with CTL 495 to the same level as the Tp9_67–75_ Muguga epitope. This experiment identified position 5, 7, and 8 to be important for TCR recognition by both Tp9-specific CTL lines and position 6 as important for CTL 4003 only (Fig. 3A). Because the cell line CTL 495 is heterozygous (A10/A14), it is theoretically possible that the observed difference for Tp9_A2_ and Tp9_A6_ between the two cell lines could be because of additional MHC alleles from the A10 haplotype of CTL 495 that bind the peptide. However, this was ruled out by using the Net MHC pan 4.1 version (https://services.healthtech.dtu.dk/service.php?NetMHCpan-4.1) to predict binding of the peptide for the two MHC alleles from the A10 haplotype (BoLA-2*012:01 and BoLA-3*002:01).

To confirm this pattern, we generated peptide–MHC class I Tets using all peptides that could bind to the BoLA-1*023:01 class I molecule, to assess their binding capacity by flow cytometry (Fig. 3B). We used CTL 4003 for this evaluation and observed that Tets containing Tp9_A5_ to Tp9_A8_ peptides were not able to stain the CD8^+^ CTL, confirming that each of the amino acids in these positions is required for TCR recognition by this CTL.

### Determination of the ability of naturally occurring variants of the Tp9 epitope to bind BoLA-1*023:01

**FIGURE 4.**
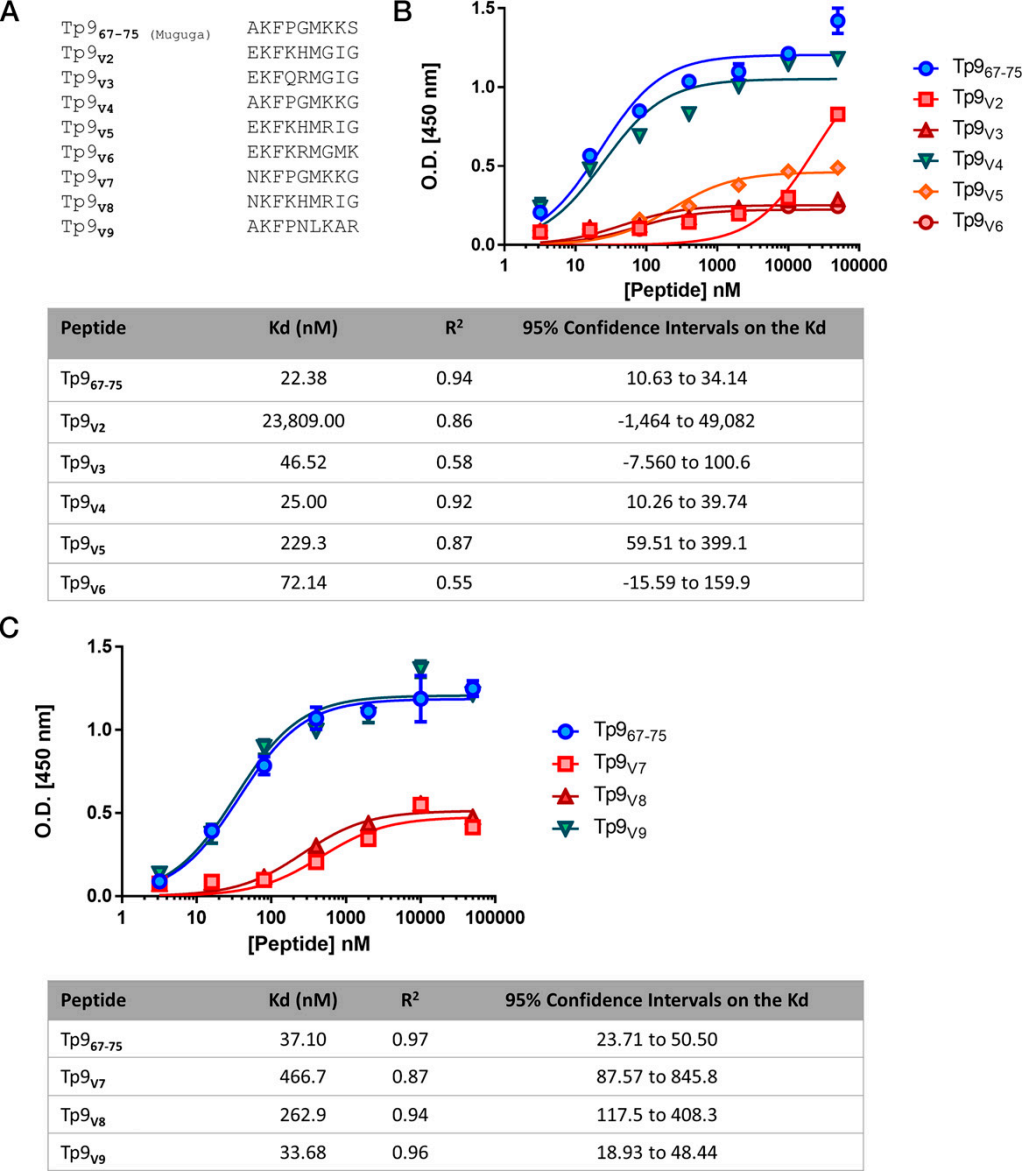
Binding affinities of Tp9 field variants. (**A**) Field variants were selected from a list of published buffalo-derived and cattle-derived Tp9 sequences. The 9-mer sequences from the field variants, corresponding to the known BoLA-1*023:01–restricted epitope region of the Muguga strain, were aligned with the Tp9_67–75_ Muguga epitope sequence. (**B**) Peptide–MHC class I monomer formation ELISA using Tp9 peptide epitope sequences from these field variants: Tp9_67–75_ Muguga, Tp9_V2_, Tp9_V3_, Tp9_V4_, Tp9_V5_, and Tp9_V6_. *K*_d_ values (nM) are depicted in the table below. (**C**) Peptide–MHC class I monomer formation ELISA using Tp9 peptide epitope sequences from these field variants: Tp9_67–75_ Muguga, Tp9_V7_, Tp9_V8_, and Tp9_V9_. *K*_d_ values (nM) are depicted in the table below. Mean and SEM of a representative experiment are shown. The experiment was repeated twice with duplicate measurements.

The results using the alanine-substituted peptides raise the question of how field variants of the Tp9 epitope (Fig. 4A) would perform in binding to BoLA-1*023:01. Using the ELISA-based MHC class I binding assay, it was observed that field variants V4 and V9 retain their binding affinities to this BoLA class I molecule because their *K*_d_ values are similar to the Tp9_Muguga_ *K*_d_ value (25.00 and 33.68 nM, respectively, compared with 29.74 nM on average for Tp9_67–75_ Muguga in these assays). In the case of the field variants V3, V5, V6, V7, and V8, their binding affinity was substantially reduced, indicating that they can be considered as weak binders (46.52 nM for Tp9_V3_, 229.3 nM for Tp9_V5_, 72.14 nM for Tp9_V6_, 466.7 nM for Tp9_V7_, 262.9 nM for Tp9_V8_). On the other end of the spectrum, the Tp9 field variant V2 had an extremely higher *K*_d_ value, indicating a complete loss in binding affinity to BoLA-1*023:01 class I molecule (23,809.00 nM).

### Determination of the ability of naturally occurring variants of the Tp9 epitope to stimulate Tp9-specific CTLs

**FIGURE 5.**
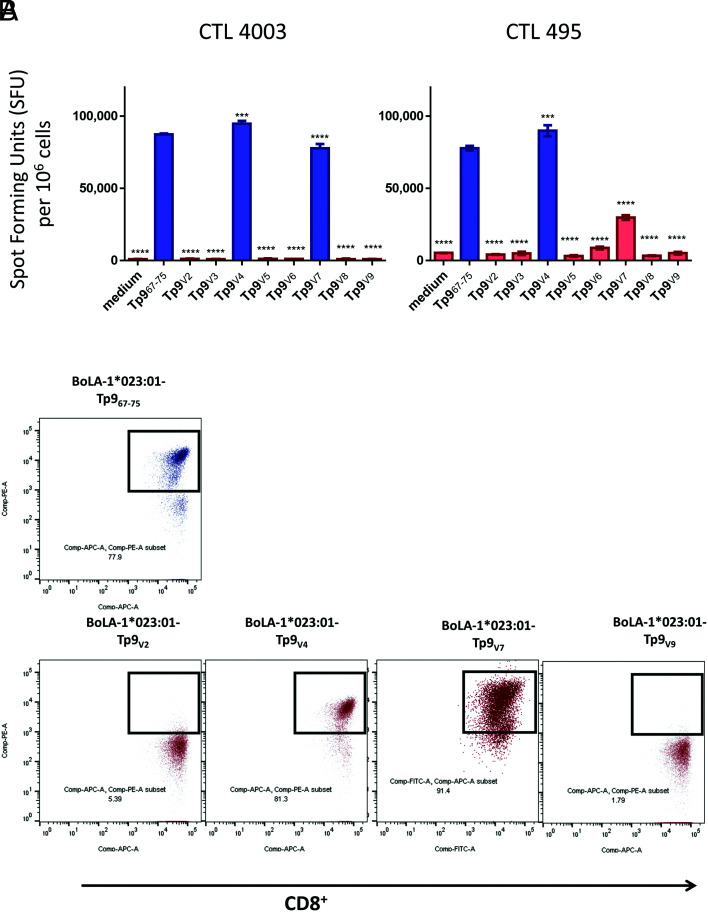
TCR recognition of Tp9 field variants. (**A**) Two Tp9-specific CTLs (from animals 4003 and 495) were subjected to an ELISPOT assay using field variant Tp9 epitopes. Data represent spot-forming units (SFUs) per 10^6^ CD8 cells. (**B**) Flow cytometry staining of CTL line 4003 with peptide–MHC class I Tets prepared with BoLA-1*023:01-Tp9_67–75_ Muguga, Tp9_V2_, Tp9_V4_, Tp9_V7_, or Tp9_V9_ and anti-CD8 Ab (ILA51). Blue histogram: CTL response maintained; red histogram: CTL response reduced. Mean and SD of a representative experiment are shown. Statistical analyses were done with Tp9_67–75_ as the reference group. A response was considered as reduced when the SFU was >15% lower than that of the Tp9_67–75_ SFU. ***p ≤ 0.001, ****p ≤ 0.0001. The experiments were repeated twice with duplicate measurements.

To evaluate whether the CTL lines were able to cross-react with variants of the Tp9 epitope, we carried out an ELISPOT assay (Fig. 5). The Tp9_V4_ variant was recognized by both Tp9-specific CTL lines to the same level as the Tp9_67–75_ Muguga epitope as measured by IFN-γ secretion. This was expected because this variant possesses only one amino acid substitution at position 9, which was shown not to be important for recognition. However, the other Tp9 variant that retained its binding capacity to BoLA-1*023:01, Tp9_V9_, was not recognized by either of the Tp9-specific CTL lines. Surprisingly, Tp9_V7_ was recognized by both CTL lines despite the lower affinity of the peptide to the BoLA, however at varying levels (Fig. 5A). This could be explained by the reduction, but not complete loss, of affinity of this peptide, causing some binding and folding of the BoLA molecule, and hence binding to the TCR. CTL 4003 was again used to confirm these observations by flow cytometry analysis of Tp9_67–75_ variants BoLA-1*023:01 class I Tet staining, and both Tp9_V4_ and Tp9_V7_ were validated as being able to bind to a Tp9_Muguga_-specific CTL line (Fig. 5B).

### Development of a TCR avidity assay using the Tp9 epitope and naturally occurring variants V4 and V7 of the epitope

**FIGURE 6.**
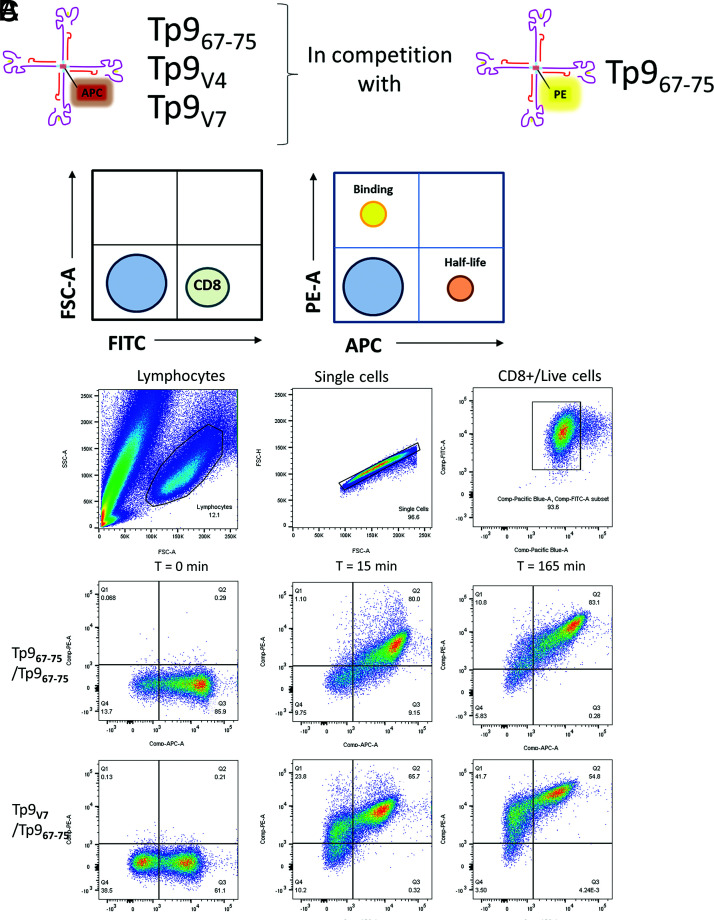
TCR avidity experimental design and example of flow cytometry gating. (**A**) Experimental setup of dual Tet competition assay to measure TCR avidity. (**B**) Example of a theoretical gating and data acquisition to measure half-life and binding of Tp9-BoLA-1*023:01 Tets with Tp9-specific CTL lines. (**C**) Example of an actual gating of single-stained allophycocyanin-positive cells (Q3, for measuring half-life) and PE-positive cells (Q1, for measuring binding) at time 0, 15, and 165 min with Tp9_67–75_ and Tp9_V7_ Tets in competition with Tp9_67–75_ Tet.

**FIGURE 7.**
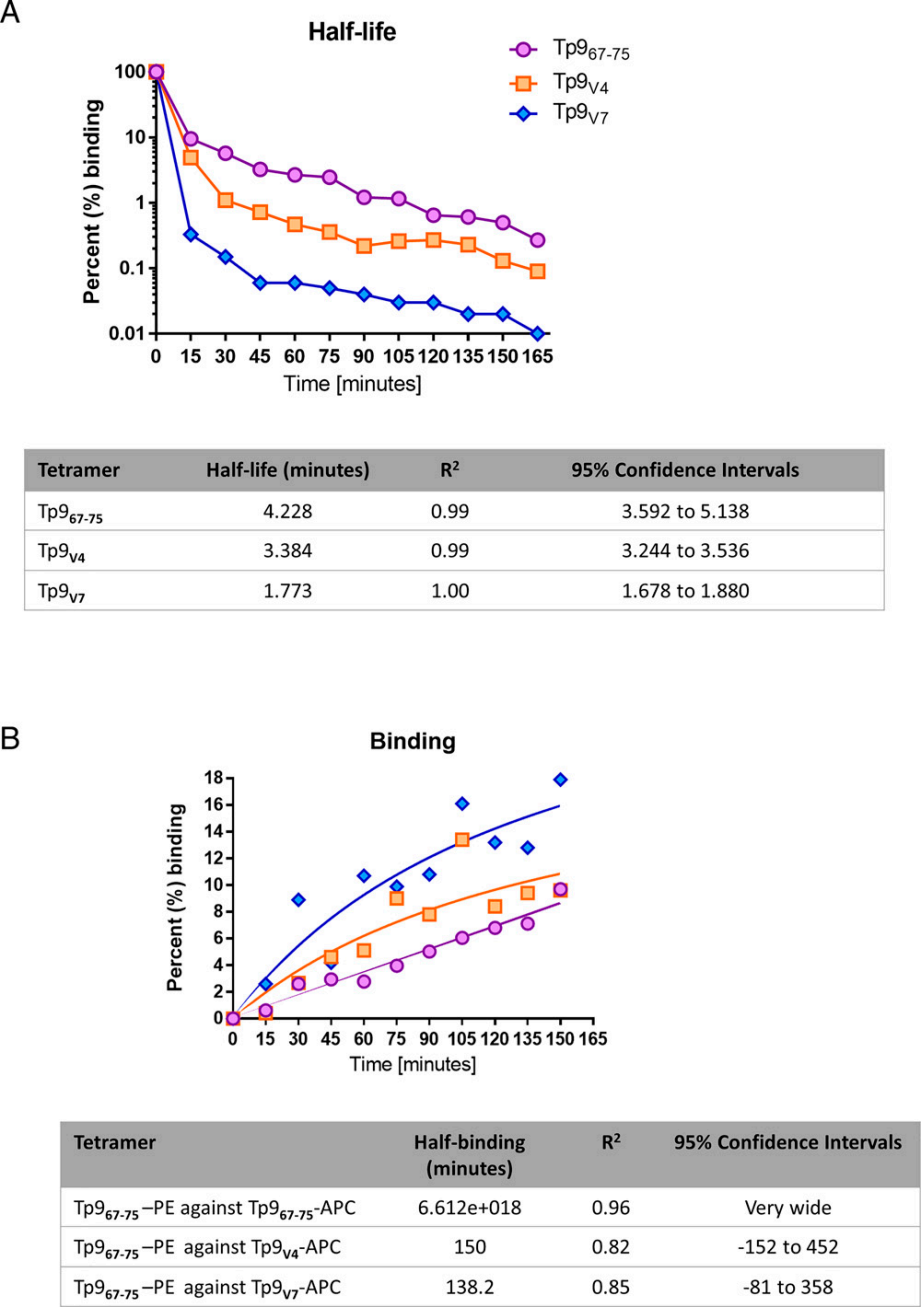
TCR avidity of CTL for Tp9 field variants. (**A**) Half-life measurements of Tp9_67–75_ Muguga, Tp9_V4_, or Tp9_V7_ Tets bound to CTL line 4003 in competition with Tp9_67–75_ Muguga. (**B**) Binding rate measurements of Tp9_67–75_ Muguga Tet competing with Tp9_67–75_ Muguga, Tp9_V4_, or Tp9_V7_ Tets bound to CTL line 4003. Half-life and binding time values (minutes) are depicted in the tables below. Values of a representative experiment are shown (at least 25,000 gated events from the lymphocytes gate were used). The experiment was repeated twice.

An assay for measuring the avidity of the binding between CTL 4003 and the Tp9 epitope or naturally occurring variants V4 and V7 of the Tp9 epitope Tets was developed. The assay was based on staining of the CTL 4003 line with allophycocyanin fluorochrome-labeled peptide–MHC class I Tets with bound Tp9 variants in competition with excess Tp9_67–75_ Muguga–MHC class I Tet coupled to a different fluorochrome, PE (Fig. 6A). We made sure that the excess Tp9_67–75_ Muguga–MHC class I Tet did not bind nonspecifically cells, when used in excess, by staining a Tp1-specific CTL line using the same conditions as with the Tp9-specific CTL line, except that the first Tet used was the Tp1-BoLA-6*013:02 Tet (Supplemental Fig. 2). Then, samples were collected at different time points over a period of 165 min and washed, fixed, and analyzed by flow cytometry to determine the half-life for the staining Tet and the binding time for the competing Tet (Fig. 6B). Only 100% allophycocyanin-stained or 100% PE-stained populations were considered in calculating the values. Double-positive cells were not factored in because we were interested in only competing Tets (PE) that had completely dislodged the staining Tet (allophycocyanin) (Fig. 6C). Using this assay, the Tp9_V7_ showed a rapid half-life in the presence of excess Tp9_67–75_ Muguga Tet (Fig. 7A). The half-life rate for the Tp9_V4_ was closer, but not equal to, what was observed for the Tp9_67–75_ Muguga Tet. This translates to a faster binding rate for the Tp9_67–75_ Muguga Tet in the presence of the Tp9_V7_ Tet, and to a lesser extent in the presence of the Tp9_v4_ Tet, but in a much slower binding rate when Tp9_67–75_ competes with itself (Fig. 7B).

### IFN-γ ELISPOT activity in the presence of varying concentrations of the Tp9 epitope and naturally occurring variants V4 and V7 of the epitope

**FIGURE 8.**
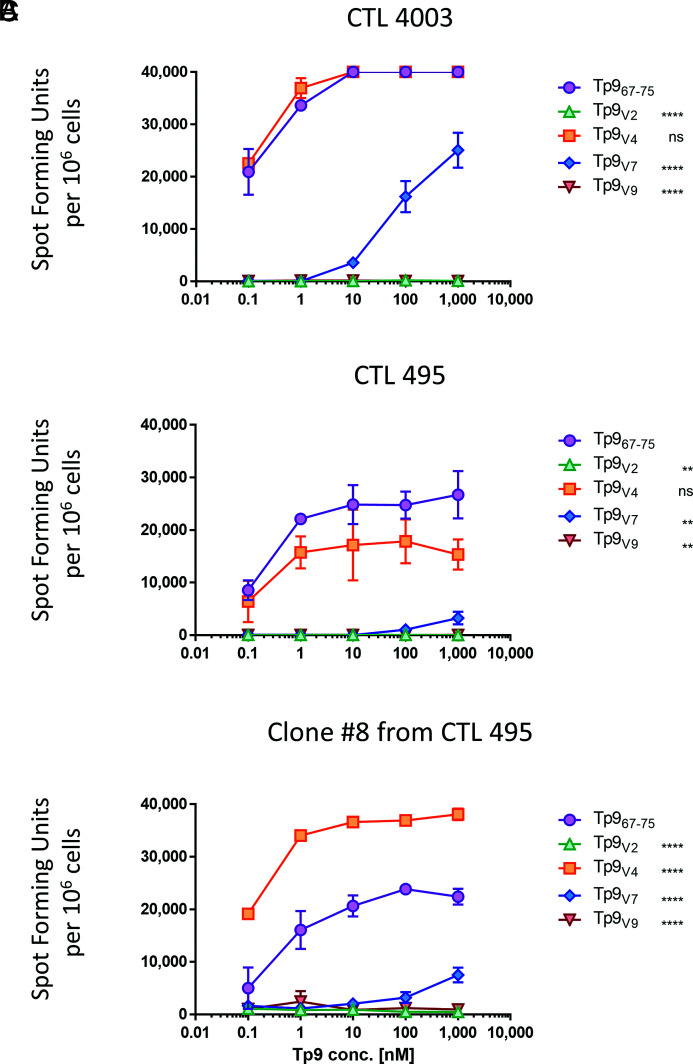
Reactogenicity determination of Tp9-specific CTL lines toward different concentration of Tp9 field variant epitopes. (**A**) ELISPOT assay with CTL 4003. (**B**) ELISPOT assay with CTL 495. (**C**) ELISPOT assay with a clone (clone 8) of CTL 495 with peptides ranging from 0.1 to 1000 nM. Mean and SD of a representative experiment are shown. ^ns^*p* > 0.05, **p ≤ 0.01, ****p ≤ 0.0001. The experiment was repeated twice with duplicate measurements.

**FIGURE 9.**
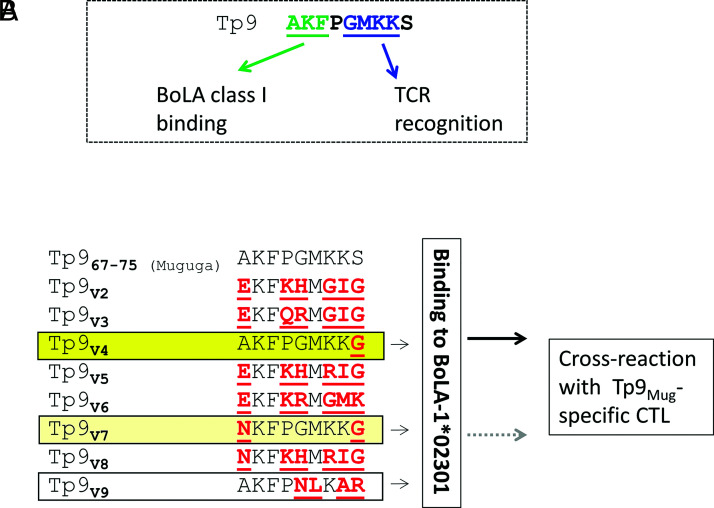
Binding affinity and TCR recognition of Tp9 in relation to cross-protection. (**A**) Summary schematic presentation of anchor positions and TCR recognition sites on the Tp9_67–75_ Muguga epitope. The green color indicates the amino acids in the Tp9_Muguga_ epitope important for binding to the BoLA-1*023:01 molecule, and the blue color indicates the amino acids important for TCR recognition. (**B**) Summary of BoLA-1*023:01 binding and Tp9-specific TCR recognition by the field variants of Tp9.

To underpin the observed variation in binding kinetics between the Tets, we conducted an ELISPOT assay with the two Tp9-specific CTLs and a clone from CTL 495 (clone 8) by exposing them to Tp9_67–75_ Muguga, Tp9_V4_, and Tp9_V7_ at varying concentrations. CTL 4003 was stimulated equally well by Tp9_67–75_ Muguga and Tp9_V4_ epitopes (Fig. 8A), whereas CTL 495 showed a higher sensitivity to the Tp9_67–75_ Muguga epitope compared with Tp9_V4_, for which the response was slightly decreased (Fig. 8B). However, using a clone of CTL 495, a much higher sensitivity was observed toward the Tp9_V4_ epitope as compared with the Tp9_67–75_ Muguga epitope, which was the original epitope, used for generation of this CTL line (Fig. 8C). In all assays using the two CTL lines and the clone, cells showed very little reactivity to Tp9_V7_, except for CTL 4003 at higher peptide concentrations (Fig. 8), confirming our observation using the TCR avidity assay. These observations indicate that the V4 and V7 variants are the only ones, among the variants tested, to have the capacity to bind to BoLA-1*023:01 and to stimulate Tp9MUGUGA-specific CTLs (Fig. 9).

## Discussion

The development of an effective subunit vaccine against *T. parva* will require a thorough understanding of the protective cellular immune response to the parasite and identification of the correct panel of Ags that can provide broad protection against different field strains. A detailed characterization of the ability of *T. parva*–specific cytotoxic CD8^+^ T lymphocytes to recognize variants of these Ags from parasite strains in different endemic areas is needed to achieve this. This study was undertaken to investigate the ability of Tp9 epitope–specific CTLs restricted by BoLA-1*023:01 to cross-react with antigenic variants from other parasite strains.

Previous studies have demonstrated that partial protection can be achieved against challenge with a homologous strain of the parasite (33). Furthermore, early studies showed some level of interstrain cross-protection, and more recent studies indicated that immunizing cattle with the Muguga strain of *T. parva* resulted in substantial cross-reactivity of the CTLs with the parasite strains included in the Muguga cocktail (Serengeti-transformed and Kiambu 5) and with other selected strains (45). This indicated that there are epitopes in *T. parva* leading to CTL cross-reaction. Nonetheless, recent studies have shown that differential specificities toward CTL Ags can emerge from immunization with the Muguga cocktail ITM vaccine, even in identical MHC background (46), and breakthroughs have been observed in the field with cattle immunized by ITM in a buffalo-endemic area (47). Moreover, partial cross-reaction of a Tp2 CTL line toward certain natural variants of the Tp2_49–59_ *T. parva* CTL epitope has been demonstrated in vitro (48). However, the majority of these variants were escape mutants.

An immune response toward Tp9 in ITM-immunized cattle has been demonstrated (49), and its equivalent in *T. annulata*, Ta9, has been shown to be an immunodominant Ag for the CTL response in BoLA-A14 cattle (50, 51). Remarkably, the CTL response in such cattle is directed to a single epitope on Tp9, which is restricted by the BoLA-1*023:01 allele and is one of the most prevalent BoLA class I alleles in Kenya (N. Svitek, unpublished observations). However, the *T. parva* Tp9 Ag and Tp9 epitope have recently been shown to be polymorphic in field strains, which could potentially reduce the efficacy of Tp9 in the design of subunit vaccines. We found that CTLs with specificity to Tp9_Muguga_ recognized only two of the eight-field variant Tp9 epitopes, suggesting that this would in fact be problematic if Tp9 is used as a CD8^+^ T cell Ag in the context of the BoLA-1*023:01 class I molecule. The cross-reactivity in our study is done from the perspective of the *T. parva* Muguga strain, which is a limitation. However, it is the only strain for which CTL epitopes have been discovered, and it is likely that only the strains behind Tp9_V4_ and Tp9_V7_ could elicit CTL to the same epitope from *T. parva*. Because we did not have these strains isolated and produced as stabilates for immunization, this was not possible to investigate. However, this Ag may still be used as a promising cellular immune Ag because it induces CD4^+^ T cell responses in several MHC class II haplotype backgrounds (49, 52). Alternatively, several identified *T. parva* CTL Ags, such as Tp5, which are not polymorphic or show very little polymorphism, could be combined with Tp9 to induce broad CD4^+^ and CD8^+^ protective T cell responses.

Binding of peptides to MHC class I molecules is a selective process in the presentation of epitopes to T lymphocytes. Insights into the mechanisms that influence peptide binding to MHC class I molecules can provide a better understanding of factors important for triggering an optimal cellular immune response and of the initial events that govern T cell function. In this study, positions 2 and 3 in the Tp9 epitope were identified as anchor positions from the alanine scanning experiments. Interestingly, the reduction in binding of the peptides harboring an alanine substitution in positions 2 and 3 is consistent with the elution data obtained from a recent mass spectrometry study of peptides eluted from BoLA molecules of a A14-haplotype TpM line, which indicated that these two positions are favored by BoLA-1*023:01 (41). Moreover, even though position 9 is of importance for BoLA-1*023:01, based on the published mass spectrometry data (41), the serine at this position in the Tp9 epitope is not among the amino acids that are preferred by BoLA-1*023:01, which include leucine (L), phenylalanine (F), tyrosine (Y), and methionine (M) (Supplemental Fig. 3A). Therefore, the data obtained with the alanine substitution at position 9 of Tp9 is again consistent with the elution data. Moreover, replacing the serine with an alanine at position 9, which should have a slight detrimental effect on binding of the epitope according to the elution data, does not affect binding of Tp9_67–75_ and confirms that this position is not required in the context of the Tp9_Muguga_ epitope. In addition, a previous study was performed to determine the anchor residues in 9-mer peptides binding to BoLA-1*023:01 using positional scanning combinatorial peptide libraries (PSCPLs) (53). The PSCPLs found a binding motif with the anchor position 9 as the most preferred by this MHC class I molecule, with methionine (M), phenylalanine (F), tyrosine (Y), histidine (H), or leucine (L) being again the amino acids of choice at this position (Supplemental Fig. 3B). According to this study, the binding motif of BoLA-1*023:01 had other anchor positions of lesser importance, which appeared to be present at positions 1, 2, and 6. Interestingly, the two residues of Tp9 that were required for binding to BoLA-1*023:01 based on the alanine scanning were K and F at positions 2 and 3 (Fig. 9A). Corroborating this observation, in the mass spectrometry study, a lysine has been shown to be highly prevalent at position 2 of 9-mer peptides (mostly self-peptides) eluted and analyzed by mass spectrometry from BoLA molecules of a A14-haplotype TpM line (41). Most of the field variants had reduced binding affinities for BoLA-1*023:01, even though they had the K and F intact at positions 2 and 3, indicating that other positions influenced the binding. However, variant 2 (Tp9_V2_) completely lost the binding capacity (23,809.00 nM) (Fig. 9B). The reduction in binding of field variants is most probably due to the presence of several amino acid substitutions that may cause overall conformational changes of the peptide and possibly distort the orientation of the lateral chains of the amino acids at key anchor positions. This could be the result of polar amino acids that contain aromatic rings, such as the histidine, or positively charged amino acids with long lateral chains, such as the arginine or the tyrosine, at positions 4 and 5, which are neighboring the anchor positions 2 and 3 in most of the field variants and could alter their conformation. Furthermore, the difference in binding between variants 4 (25.00 nM) and 7 (466.7 nM) is most probably due to the unique difference between the two variants at position 1, where in the former the alanine is intact as compared with Tp9_Muguga_ and in the latter the alanine is substituted with an asparagine. This seems to have an impact in the binding of variant 7 because it reduces the binding affinity to the BoLA molecule to similar levels as measured with the Tp9_A2_ and Tp9_A3_ peptides and the TCR avidity of the peptide–BoLA complex. This indicates that the asparagine at this position has a detrimental effect on the binding of this variant. This is in agreement with data generated by the PSCPL experiments using random 9-mer peptides, which indicated that the asparagine in position 1 had a detrimental effect on peptide binding in the peptide binding groove of the BoLA-1*023:01 molecule. This suggests that position 1 of this Tp9 epitope also plays a role in binding and can be considered an anchor position in the context of the Tp9_Muguga_ epitope (Fig. 9A).

Recent reports have shown a better correlation of peptide–MHC class I stability assay with the antigenic quality of an epitope (54), which measures the rate at which the MHC class I molecule unfolds in the presence of an immunodominant epitope, compared with the binding assay that measures the lowest concentration needed to achieve proper folding of the MHC class I molecule on binding by an immunodominant epitope. The stability assay could therefore be included in future studies to characterize epitope binding. However, the requirement of using radioactive isotopes impairs the accessibility and practicality of these assays.

In the case of the TCR recognition of the Tp9 epitope, the amino acids at positions 5–8 (GMKK) all seem to be important because only variants 4 and 7, which have a GMKK sequence intact in this region, retain their capacity to stimulate Tp9_Muguga_-specific CTL lines (Fig. 9B). An interesting observation was that some alanine-substituted Tp9_Muguga_ peptides had an increased binding affinity to the BoLA molecule. This resulted in an increased reactivity by the Tp9-specific CTL lines toward these mutant peptides. For instance, the Tp9_G1_ and Tp9_A9_ peptides had a higher binding affinity to BoLA-1*023:01 class I molecule, which resulted in an enhanced stimulation of CTL 4003 and 495 in the ELISPOT assay (one-way ANOVA with Dunnett’s correction for multiple comparison, *p* ≤ 0.01 and *p* ≤ 0.001, respectively). Some studies have shown the impact of replacing amino acids at key positions in a T cell epitope for the improvement of MHC class I stability, kinetics, and immunogenicity (55, 56). This observation indicates that epitope or Ag engineering of the Tp9 epitope region could potentially lead to a stronger Ag and potentially increase cross-reactivity with other field strains. The difference in response between the CTL lines toward the different Tp9 alanine mutant peptides and field variants is not an unusual phenomenon. A previous study from our group had observed that CTLs from cattle expressing the same BoLA class I molecules react differently toward the same *T. parva* strain (46). These differences can be caused by several factors, including differences in TCR repertoire, single-nucleotide polymorphism in innate or adaptive immunity genes, or difference in epigenetic factors.

It is largely accepted that a stronger TCR avidity correlates with a better recognition of infected cells (57, 58), and the relationship between avidity and TCR functionality has been the subject of intense research to improve T cell–based immunotherapy against cancer (59). Furthermore, the TCR of memory Th1 cells has a reduced requirement for the involvement of stimulation by costimulatory molecules because they become more sensitive on engagement of their TCR by an infected cell (60), suggesting a direct correlation of TCR avidity with a rapid immune response. Moreover, a stronger TCR affinity, which measures the strength of the interaction between a single TCR and a peptide–MHC molecule and contributes to the overall avidity of several TCRs toward p-MHC complexes during the formation of an immunological synapse, has been correlated with the polyfunctionality of CD8^+^ T cells (61) and therefore with a better functional response to pathogens. A stronger TCR affinity has also been correlated with a swifter response to an infected cell, while a lower-affinity TCR–antigen interaction requires more time to elicit the same response (62). The high sensitivity of a TCR molecule is of particular value for the detection of low-intensity Ags, such as those derived from intracellular pathogens, which can downregulate p-MHC complexes on the surface of infected cells. However, above a specific TCR–p-MHC threshold, T cell function cannot be enhanced (57), and in some cases, it can be detrimental to T cell functionality (63). The ability to measure TCR avidity can help in selecting the best epitope, and several assays have been developed to measure the avidity of TCRs toward their epitopes, such as Tet dilution assay (64), the use of MHC class I–specific Abs that compete with Tet binding (65), or the use of functional cellular assays such as ELISPOT or cytotoxicity assays. In this study, we developed and optimized an assay to measure both half-life and binding rates of peptide–MHC class I to TCR. One of the advantages of the assay is that inhibitory mAbs are not needed. All that is needed is the same Tet generated with two different fluorochromes.

In summary, we have identified key amino acids that are required for BoLA class I and TCR recognition by the polymorphic Tp9_67–75_ epitope. To our knowledge, this is the first report to study the impact of Tp9 polymorphism on binding to BoLA class I molecules and recognition by a Tp9_Muguga_-specific T cell line and stress the importance of understanding the impact of CTL Ag polymorphism in the immunobiology of *T. parva*. This study also describes an easy-to-use TCR avidity assay for the characterization of the cross-reactivity by T cells toward variant epitopes from *T. parva* field strains. These results suggest that Tp9 alone would not be sufficient for covering BoLA A14–positive cattle in a subunit vaccine because of polymorphisms, and that other Ags would be needed to provide broad-spectrum immunity toward *T. parva* strains circulating in the region.

## Supplementary Material

Data Supplement
