# Supplementary material for: Systematic Determination of TCR–Antigen and Peptide–MHC Binding Kinetics among Field Variants of a Theileria parva Polymorphic CTL Epitope
Source: J Immunol. 2022 Feb 1;208(3):549–61. doi: 10.4049/jimmunol.2100400 (PMC8802549; doi:10.4049/jimmunol.2100400)
Supplement: Data Supplement [file JI_2100400.zip › JI_2100400_Supplemental_1.pdf]

Supplemental Figure 1.

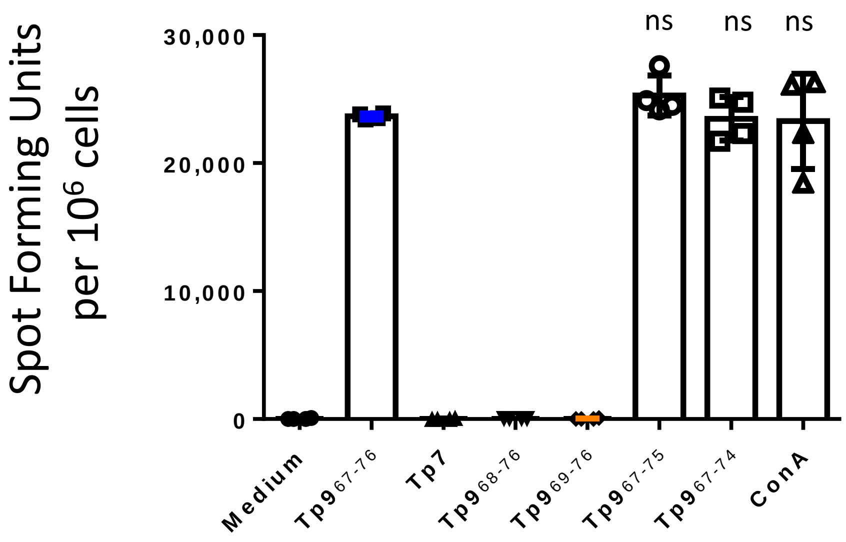

Supplemental Figure 1. ELISpot assay with Tp9 minimal epitopes and a Tp9-specific CTL line (495). Peptide concentration used: 1 μM. ConA: Concanavalin A – stimulated positive control. ns: non-significant.

Supplemental Figure 2.

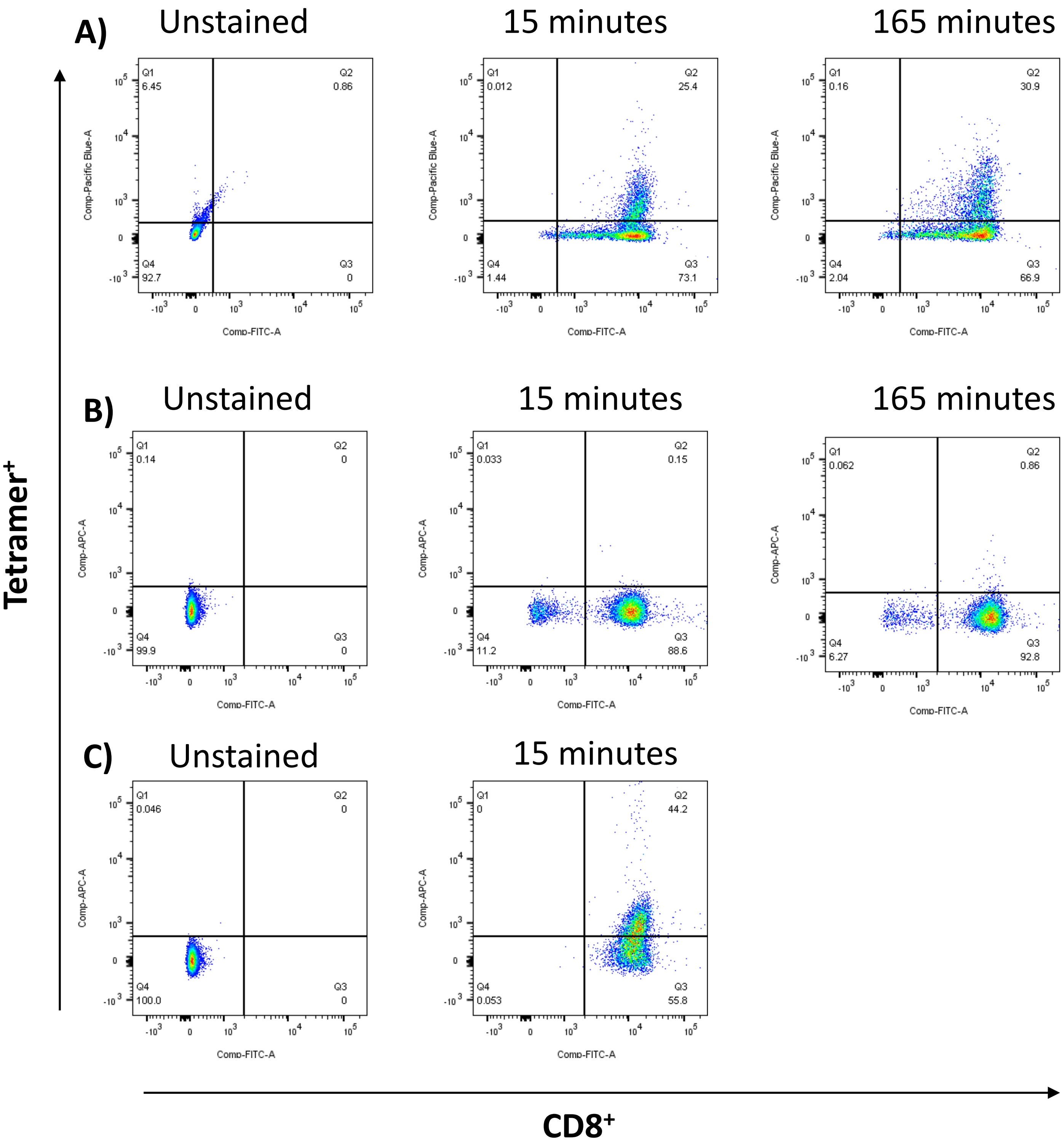

Supplemental Figure 2. Flow cytometry assay of Tp1-specific CTL (BF092) stained with excess Tp9$_{67\text{-}75}$-BoLA-6*01302 tetramer. A) BF092 CTL line showing staining with first Tp1-BoLA-6*01302 tetramer. **B)** BF092 CTL line showing staining with second Tp9-BoLA-1*02301 tetramer used in excess. **C)** 495 cell line (Tp9-specific) stained with Tp9-BoLA-1*02301 tetramer.

Supplemental Figure 3.

A)

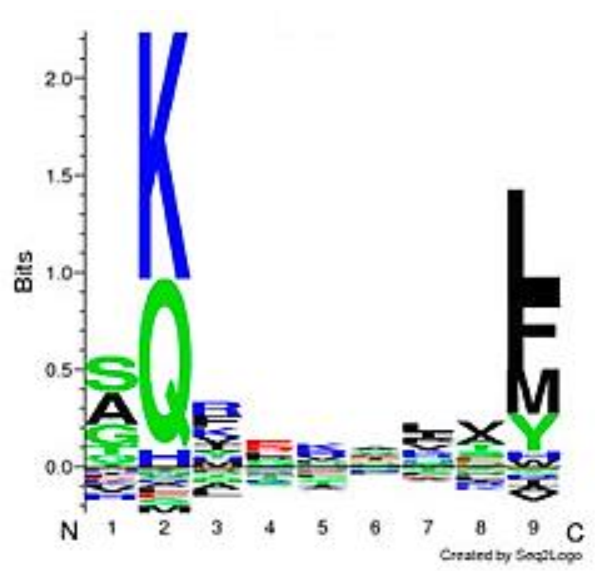

B)

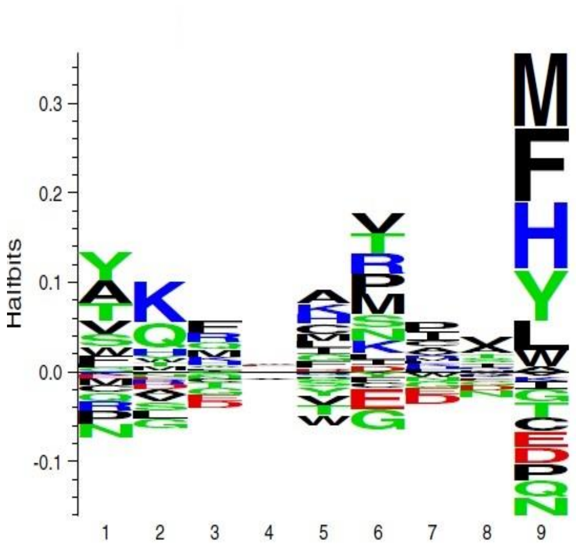

Supplemental Figure 3. Sequence logo representation of the binding motif of the BoLA-1*02301. A) Sequence from the published BoLA elution data (40). **B)** Sequence from the published PSCPL data (51).
